# Impact of Negative Symptom Domains and Other Clinical Characteristics on Functional Outcomes in Patients with Schizophrenia

**DOI:** 10.1155/2021/8864352

**Published:** 2021-02-20

**Authors:** Hiroki Okada, Daisuke Hirano, Takamichi Taniguchi

**Affiliations:** ^1^Department of Occupational Therapy, Medical Corporation Nasukougen Hospital, Nasu, Tochigi, Japan; ^2^Department of Occupational Therapy, School of Health Science, International University of Health and Welfare, Ōtawara, Tochigi, Japan

## Abstract

Negative symptoms of schizophrenia have generally been defined using five factors; however, few studies have examined the relationship between these five factors and functional outcomes. In addition, there is no definitive conclusion regarding the association between negative symptoms and various aspects of functional outcomes (daily living, social, and vocational). This study is aimed at examining the relationship between these five domains of negative symptoms and different functional outcomes. Patients diagnosed with chronic schizophrenia (*n* = 100) were selected for the evaluation. We used the Brief Negative Symptom Scale to assess negative symptoms, the Brief Psychiatric Rating Scale to assess positive symptoms, the Schizophrenia Cognition Rating Scale to assess cognition, and the Evaluative Beliefs Scale (negative self-assessment) to assess psychological factors. We analyzed their relative impact on Social Functioning Scale domains using hierarchical multiple regression analysis. Concerning the relationship between daily living and negative symptoms, cognitive function showed the highest association with residential outcomes, such as self-care and shopping, while avolition appeared to show an additional contribution; however, for recreational outcomes, avolition showed the main association, whereas cognitive function showed no additional contribution. For social outcomes, asociality and negative self-assessment showed the main associations, while vocational outcomes were determined by both cognitive function and multiple negative symptoms, such as avolition, anhedonia, asociality, and alogia. Since negative symptom domains appear to differentially impact each outcome, specifically daily living outcome, it is important to evaluate the residential outcomes and recreational outcomes separately. Overall, the present study points to the importance of formulating psychosocial treatment strategies specific for each type of preferred outcome in patients with schizophrenia.

## 1. Introduction

Functional disabilities are a central characteristic of schizophrenia [1]. Therefore, researchers have focused on identifying factors that contribute to functional outcomes in schizophrenia, allowing the development of new treatment methods. Previous research has identified that certain clinical factors may create barriers for individuals with schizophrenia, limiting their daily activities, as well as their social and vocational potential [2]. Among these factors, negative symptoms have emerged as key predictors of functional outcomes in individuals with chronic schizophrenia [3]. There is mounting evidence that the negative symptoms of schizophrenia, defined as the absence or diminution of normal behavior and function, are core features of the disorder even during the first episode. Like cognitive deficits, these negative symptoms have prognostic importance and appear before the emergence of positive symptoms [4]. Despite seminal scientific advances in genetics, biology, psychopharmacology, epidemiology, diagnosis, and schizophrenia treatment, negative symptoms have proven to be resistant to current psychopharmacological treatments. Treating negative symptoms in patients with schizophrenia in order to achieve sustained periods of remission can be challenging [5]. Among other reasons, the multidimensional aspect of negative symptoms complicates this type of research. Therefore, it is necessary to prioritize research in these particular areas, specifically to clarify the complex association between negative symptoms and functional outcomes.

As described in the National Institute of Mental Health (NIMH) report [6], negative symptoms are categorized as experience- and expression-based factors. Experience factors in individuals with schizophrenia are associated with a lack of motivation and ability to enjoy pleasant life experiences, and they include anhedonia (inability to experience joy), asociality (decreased value of social contacts), and avolition (decreased motivation). Expression factors indicate a decline in linguistic and nonverbal communication, including blunted affects (decrease in nonverbal emotional expression) and alogia (decrease in the amount of speech). Recent studies, using a two-dimensional model, have investigated the effects of these two negative symptom factors (experience and expression factors) on functional outcomes [7, 8]. However, several factor-based analytical studies suggest that a two-dimensional model is insufficient, and large-scale studies support the validity of a five-factor structure of negative symptoms [9, 10]. Studies using a two-dimensional model showing that expression- and experience-based factors are predictive of functional outcomes might not be capturing the complexity and specificities of these associations. For example, the data from studies using a two-dimensional model suggest that experience factors can predict the functional outcomes. By contrast, other studies using a two-dimensional model suggest that expression factors (such as blunted affect) are more important predictors of functional outcomes [11]. The discrepancies among the studies using the two-dimensional model suggest that a more specific five-domain model may be required to robustly analyze the negative symptoms.

The multidimensional aspect of functional outcome evaluations also complicates the analyses. Since functional outcome scales that generate global scores and collapse across functional domains may give an incomplete, or even misleading, perspective on overall outcomes, recent studies have analyzed them by dividing them into daily living, social, and vocational outcomes [12]. Large-scale studies assessing these outcomes have revealed different determinants of daily living, social, and vocational outcomes [13–18]. For example, negative symptoms appear to be consistently related to social outcomes, with additional contributions from cognitive function and ability [13–16]. In addition, for vocational outcomes, there is a consistent association among experiential factors, with cognitive function and ability being also greatly affected [14–16].

In contrast, no conclusions have been reached regarding the relationship between negative symptoms and daily living outcomes. In some large-scale studies, deficits in daily living outcomes were poorly associated with negative symptoms as compared to the social and vocational outcomes [13–16]. Contrary to the results of these large-scale studies, several cross-sectional and cohort studies have suggested a strong association between negative symptoms and daily living outcomes [17, 18]. In particular, Strassnig et al. showed that negative symptoms are the predictors of daily living function [18]. These discrepancies may be due to the methodological differences and the lack of specificity of the models used to evaluate negative symptoms. In these studies, the scale for assessing negative symptoms had a small number of items and did not properly evaluate each domain [19]. Therefore, Kirkpatrick et al. developed the Brief Negative Symptom Scale (BNSS), which adequately assesses domains with negative symptoms [19]. Recent studies using BNSS reported that among the negative symptoms' subdomains, avolition explains 30% of the variance in functional outcomes [20]. Specific aspects for functional outcomes, however, were not mentioned. In the present study, we employed the BNSS to assess the five domains of negative symptoms, along with the Social Functioning Scale (SFS) [21], allowing for a multifaceted evaluation of the functional outcomes. The relative impact of the five domains of negative symptoms and other clinical factors on functional outcome was investigated. Based on the prior research, we also examined the cognitive function, positive symptoms, and psychological factors, specifically related to the functional outcomes [7, 8, 13].

## 2. Materials and Methods

### 2.1. Patients

This study was approved by the Institutional Review Board of the International University of Health and Welfare. All participants provided written informed consent. This study was performed in accordance with the principles of the Declaration of Helsinki.

In total, 100 patients were recruited from the outpatient treatment clinics at the Nasukougen Hospital in Japan. Patients were selected based on an International Statistical Classification of Diseases and Related Health Problems–version 10 (ICD-10) diagnosis of schizophrenia or schizoaffective disorder. The exclusion criteria included (1) substance use disorder; (2) history of neurological disorders such as seizure, stroke, head injury, brain surgery, mental retardation, or migraine; and (3) age < 20 or >65 years.

All study participants were stable patients with schizophrenia who had not been hospitalized or readmitted to a psychiatric hospital in the past six months and had not used emergency medical services in the past year. Outpatient status was defined as living outside any institutional setting, including nursing homes.

### 2.2. Procedures

After study eligibility was determined by an intake evaluation, the participants underwent a series of structured clinical assessments and symptoms and functioning measurements. Except for functional outcomes, all symptoms were evaluated by the authors and the attending physician. Cognitive function was evaluated by the authors, responsible nurses, and health care workers.

### 2.3. Assessment

Negative symptom severity was assessed using the BNSS. Based on a recent factor analysis, positive symptom severity was assessed using a subset of the Brief Psychiatric Rating Scale (BPRS) [22], which included the following items: grandiosity, suspiciousness, hallucinations, unusual thought content, bizarre behavior, disorientation, and conceptual disorganization. Neurocognitive function was assessed using the Schizophrenia Cognition Rating Scale (SCoRS). Psychological factors were evaluated using the Evaluative Beliefs Scale (EBS). Functional outcome was measured with the SFS. All of these evaluation scales were translated into Japanese [23–27].

#### 2.3.1. BNSS

BNSS is a scale for detecting negative symptoms based on the NIMH consensus statement. It evaluates six items: anhedonia, asociality, avolition, blunted affect, alogia, and distress. However, distress was excluded from this study because it is not an agreed-upon domain by the NIMH [6]. Symptoms were scored as follows: 0 to 21 for anhedonia, 0 to 12 for asociality, 0 to 12 for avolition, 0 to 21 for blunted affect, and 0 to 12 for alogia. The Japanese version of the BNSS was employed in this study [19].

#### 2.3.2. BPRS

The BPRS was created by Overall and Gorham (1962) to evaluate a wide range of mental symptoms. In this study, the BPRS was employed to identify positive symptoms, using seven items related to positive symptoms. We used the Japanese version of the BPRS, which ranged from a score of 0 to 42 [22].

#### 2.3.3. SCoRS

The SCoRS evaluates cognitive function and the ability to perform cognitive skills in patients with schizophrenia based on the recommendations from the Measurement and Treatment Research to Improve Cognition of Schizophrenia (MATRICS) project. Seven cognitive domains were evaluated: vigilance, working memory, processing speed, language learning and memory, visual learning and memory, reasoning and problem solving, and social cognition. We used the Japanese version of the SCoRS [25], which reports scores ranging from 20 to 80.

#### 2.3.4. EBS

The EBS contains 18 items that measure global and stable negative evaluative beliefs [26]. The EBS is composed of three subscales. The range of possible scores for each scale is 0–18. The first subscale measures negative beliefs about the participant's self-image (e.g., “I think I am totally bad”). The second subscale measures negative beliefs that the participant has about other people's judgment about him or her (e.g., “Other people think I am totally bad”). The third subscale measures negative beliefs that the participant may have about others (e.g., “Other people are totally bad”). In this study, the reliability was high for all subscales (*α*¼0.87, *α*¼0.91, and *α*¼0.85, respectively). We used the Japanese version of the EBS that was translated by Furumura et al. [27].

#### 2.3.5. SFS

The SFS evaluates the various fields of functional outcomes in patients with schizophrenia. It has seven subitems: (1) withdrawal, (2) interpersonal relationships, (3) social participation, (4) recreation, (5) self-reliance and ability, (6) self-reliance and execution, and (7) employment. In the present study, the subitem of self-reliance and ability was excluded because it was evaluated as the possession of cognitive skills [21]. Daily living outcomes were evaluated by the subitems (4) recreation and (5) self-reliance and execution; these primarily assess the extent to which recreational and residential activities such as food preparation and shopping are performed. Social outcomes were evaluated by withdrawal, interpersonal relationships, and social participation; they primarily assess the quantity and quality of social participation and interpersonal interaction. Vocational outcomes were evaluated by the subitem employment, which mainly determines how much time the participant spends doing work and housework in a day and a week. The definitions of daily living, social, and vocational outcomes were based on the Specific Level of Functioning Scale, which can adequately evaluate the functional outcomes [28, 29]. We used the Japanese version of the SFS as translated by Nemoto et al. [30].

### 2.4. Statistical Analysis

To investigate the relative importance of the SFS domains, BNSS domains, and other variables, three methods were utilized. First, Pearson's product-moment correlation coefficient was used to determine the correlation between the SFS subitems, BNSS domains, and other variables. Next, a hierarchical regression analysis including the significant variables from the correlation analysis as independent variables was employed. In block 1, the variable selection was performed by the stepwise method on individual SFS domains. Furthermore, in block 2 and later, the best-subset method was used to explore the factors of individual SFS regions and to examine the optimal model. Due to the lack of prior research, we conducted an exploratory analysis. Therefore, this study did not use a multiple comparisons correction, and the significance level was set at *p* < 0.05.

In some correlations, the same characteristic can be considered as both the dependent and independent variables, which may cloud the association. Therefore, in this study, the outcome evaluators were not involved in the evaluation of social functions. Furthermore, the problem of multicollinearity of each domain was addressed by the variance inflation factor (VIF) values. Because VIF values between 5 and 10 indicate strong correlation, which may be problematic [31], the domains with a VIF value ≥ 5 were considered to have multicollinearity.

## 3. Results

Table 1 shows the patients' demographic information and the descriptive statistical parameters for each variable. Since the EBS score was missing for seven patients, the mean imputation method was used for this variable.  Table 2 shows the correlation coefficients between the SFS domains, BNSS domains, and others. All SFS domains correlated with the BNSS domains, positive symptoms, and cognitive function. By contrast, the EBS subscales showed little correlation except for negative self-evaluation. Multiple regression analysis was performed using the BNSS domains, positive symptoms, cognitive function, and negative self-evaluation as independent variables.

Table 3 shows the final results of the hierarchical regression analysis after best-subset method analysis. As for (1) withdrawal and (2) interpersonal relationships, asociality remained, and the inclusion of negative self-evaluation in the model resulted in the best-fit and a statistically significant increase in the variance explanation rate. However, among the social outcomes, only asociality remained in (3) social participation, whereas introducing additional variables did not result in a statistically significant increase in the variance explanation rate. Similarly, the results of daily living outcomes were diverse. As for (4) recreation, avolition remained, and inclusion of asociality in the model resulted in the best-fit and a statistically significant increase in the variance explanation rate. However, for (6) self-reliance and execution, cognitive function remained, and the inclusion of avolition in the model resulted in the best-fit and a statistically significant increase in the variance explanation rate. For (7) employment, avolition remained, and inclusion of cognitive function, alogia, anhedonia, and asociality in the model resulted in the best-fit and a statistically significant increase in the variance explanation rate.

The VIF values were as follows: anhedonia, 2.59; asociality, 3.11; avolition, 3.99; blunted affect, 2.9; alogia, 3.05; BPRS, 1.96; SCoRS, 3.68; EBS negative self-evaluation, 1.42; SFS withdrawal, 1.66; SFS interpersonal relationships, 1.64; SFS social participation, 2.14; SFS recreation, 2.78; SFS self-reliance and execution, 2.71; and SFS employment, 2.95. All VIF values were <5; therefore, no multicollinearity was observed.

## 4. Discussion

The hierarchical multiple regression analysis showed different symptoms to be the main factors in the analysis, depending on the SFS domain. The results of this study may reconcile the contradiction among the studies in which cognitive function is a major determinant of the functional outcome [32, 33] and those where negative symptoms are the major determinant [17, 34].

Concerning the relationship between daily living and negative symptoms, the focus of this study, cognitive function, showed the strongest association with residential outcomes (self-reliance and execution), such as self-care, preparation of meals, and shopping for daily necessities (e.g., groceries and clothing), and avolition seemed to have an additional contribution. Conversely, concerning recreational outcomes, avolition was the main associated symptom, and asociality was also observed. Daily living is generally regarded as an outcome related to the independent living [35], and depending on the functional outcome rating scale, daily living is assessed by integrating residential and recreational outcomes. For example, Specific Level of Functioning Scale, which has been used in many previous studies on determinants of functional outcomes, assesses daily living by integrating residential and recreational outcomes [8, 13–16]. Given the current state of inconclusive determinants of daily living, these two outcomes of daily living may need to be evaluated separately. Furthermore, these results may be explained by the mechanism of motivation. Avolition is considered a motivational obstacle, and one of the reasons patients with schizophrenia experience avolition is due to the disability in effort computation, which is the process of estimating the amount of effort required for achieving goal-oriented behavior [36, 37]. Due to this disability, people with schizophrenia tend to feel overburdened with the actions required for goal-oriented behavior [36]. Considering that cognitive function showed the strongest association with residential outcomes and avolition seemed to have an additional contribution, feeling overburdened may be associated with residential outcome similar to poor performance. Furthermore, considering the motivational mechanism, poor performance can further cause to remain overburdened, leading to poverty as a residential outcome. Regarding recreational outcomes, the degree of motivation, including feeling overburdened by recreational activities, is an important factor. As asociality was also connected to this outcome, the extent to which people engage in recreation in the real world seems to be influenced, in addition to motivation, by their level of interest in others.

Furthermore, asociality appears to be an important symptom associated with social outcomes, such as withdrawal, interpersonal relationships, and social participation. The finding that negative symptoms were primarily associated with social outcomes is consistent with those reported in previous studies [13–16]. Compared to the report by Mucci et al. [20], which showed that the major determinant of general functional outcomes is avolition, our finding that social outcomes are strongly associated with asociality is novel. Furthermore, negative self-assessments contributed additionally to these social outcomes. While psychological factors such as depression and anxiety have unequal impacts on social outcomes [38, 39], the psychological factors examined in the current study may contribute to social outcomes. However, other than negative self-evaluation, no other psychological factor was related to functional outcomes. Since items other than negative self-evaluation are imaginable from the others' viewpoints, the results may vary because of social cognitive impairment.

In terms of vocational outcomes, it was shown to be strongly associated with various aspects of negative symptoms such as avolition, anhedonia, alogia, and asociality, as well as cognitive function. As pointed out by Harvey [35], the factors affecting employment outcomes are a combination of daily living and social outcome factors. Similarly, in this study, most factors, such as cognitive function, avolition, and asociality, which were associated with daily living and social outcomes, were also associated with employment outcomes. Furthermore, vocational outcomes were associated not only with avolition but also with anhedonia and alogia, which affect motivation. For example, anhedonia, called as anticipatory pleasure, has been known to significantly predict the persistence of effortful behavior [40], and previous research has shown that alogia, the degree of language expression, is associated with motivation [41]. Needless to say, the ability to predict rewards and the degree of language expression may be important for maintaining employment. Because SFS employment scores increase with longer work and housework hours, a lack of motivation may affect the ability to perform lengthy tasks at work or at home.

In this study, positive symptoms could not explain functional outcomes. As indicated by the duration of illness and treatment doses in our study population, the lack of this relationship may be associated with the inclusion of chronic patients in this study and their treatment for positive symptoms. Therefore, our conclusions cannot be generalized to more severely ill patients. In addition, due to its exploratory nature, this study did not include multiple comparisons. Therefore, future studies should use a different analysis for each outcome.

Despite these limitations, the results of this study support those of the other emerging studies. In particular, we demonstrate the novel findings that cognitive function has an important association with residential outcomes in daily living, such as self-care and shopping, and that negative symptoms are strongly related to recreational outcomes in daily living. These findings may help resolve the contradictory results from previous research.

Although treatments are currently being developed to improve negative symptoms, cognitive function, and psychological factors in patients with schizophrenia, the results of our study emphasize the importance of developing individualized treatment strategies. In particular, the results of the present study show that a combination of cognitive and motivational interventions, including SST, cognitive remediation therapy, and environmental adjustment [42–44], would be useful in improving residential (e.g., self-care assistance) and vocational outcomes.

## 5. Conclusions

Negative symptom domains have different impacts on each outcome. In particular, for residential and recreational outcomes, which are daily living outcomes, the key associations were different. Therefore, these two outcomes should be evaluated separately. Moreover, the present study points to the importance of formulating psychosocial treatment strategies specific for each type of preferred outcome in individuals with schizophrenia.

## Figures and Tables

**Table 1:** Demographic information.

Variable	Mean (SD)
Age (years)	47.3 (12.3)
Years of education	12.3 (1.6)
Male sex (%)	38.0
Assisted living (%)	51
Duration of illness	18.2 (11)
Previous hospitalizations	1.6 (2.3)
Antipsychotic medication dose^a^ (mg)	497.8 (359.6)
BNSS anhedonia	6.1 (4.5)
BNSS asociality	4.3 (2.7)
BNSS avolition	3.7 (2.6)
BNSS blunted affect	4.7 (4.1)
BNSS alogia	3.3 (3.5)
BPRS positive	6.4 (5.3)
SCoRS	38.5 (11.2)
EBS negative self-evaluations	16.4 (6.5)
EBS negative other–self evaluations	15.4 (6.0)
EBS negative self–other evaluations	11.2 (4.3)
SFS withdrawal	9.9 (2.7)
SFS interpersonal relationships	7 (5.3)
SFS social participation	7.4 (7.0)
SFS recreation	16.1 (7.5)
SFS self-reliance and execution	24.66 (8.5)
SFS employment	4.5 (3.6)

^a^Chlorpromazine conversion. BPRS: Brief Psychiatric Rating Scale; BNSS: Brief Negative Symptom Scale; EBS: Evaluative Beliefs Scale; SCoRS: Schizophrenia Cognition Rating Scale; SFS: Social Functioning Scale.

**Table 2:** Correlations between SFS subitems and symptoms (R).

SFS subitems						
	Withdrawal	Interpersonal relations	Social participation	Recreation	Self-reliance and execution	Employment
Anhedonia	-0.277⁣^∗∗^	-0.452⁣^∗∗^	-0.344⁣^∗∗^	-0.462⁣^∗∗^	-0.285⁣^∗∗^	-0.265⁣^∗∗^
Asociality	-0.466⁣^∗∗^	-0.609⁣^∗∗^	-0.495⁣^∗∗^	-0.547⁣^∗∗^	-0.499⁣^∗∗^	-0.504⁣^∗∗^
Avolition	-0.413⁣^∗∗^	-0.460⁣^∗∗^	-0.399⁣^∗∗^	-0.557⁣^∗∗^	-0.577⁣^∗∗^	-0.665⁣^∗∗^
Blunted effect	-0.310⁣^∗∗^	-0.423⁣^∗∗^	-0.262⁣^∗∗^	-0.380⁣^∗∗^	-0.366⁣^∗∗^	-0.378⁣^∗∗^
Alogia	-0.318⁣^∗∗^	-0.396⁣^∗∗^	-0.259⁣^∗∗^	-0.397⁣^∗∗^	-0.375⁣^∗∗^	-0.338⁣^∗∗^
BPRS	-0.131	-0.110	-0.057	-0.135	-0.251⁣^∗^	-0.362⁣^∗∗^
SCoRS	-0.327⁣^∗∗^	-0.369⁣^∗∗^	-0.373⁣^∗∗^	-0.448⁣^∗∗^	-0.588⁣^∗∗^	-0.652⁣^∗∗^
Negative self-evaluations	-0.316⁣^∗∗^	-0.405⁣^∗∗^	-0.209⁣^∗^	-0.211⁣^∗^	-0.285⁣^∗∗^	-0.093
Negative other–self evaluations	-0.262⁣^∗∗^	-0.314⁣^∗∗^	-0.046	-0.091	-0.124	0.018
Negative self–other evaluations	-0.115	-0.033	0.054	0.011	0.063	0.115

R: Pearson's product moment correlation coefficient (⁣^∗^*p* < 0.05 and ⁣^∗∗^*p* < 0.01); BPRS: Brief Psychiatric Rating Scale; SCoRS: Schizophrenia Cognition Rating Scale; SFS: Social Functioning Scale.

**Table 3:** Hierarchical multiple regression analysis with different facets of functioning as the dependent variable (final model).

Model	Variable added	*β*	*t*	*p*	95% CI	*R* ^2^_Adj_
Withdrawal^a^	Asociality	-0.411	-4.527	0.000	-0.594~-0.232	0.241
	Negative self-evaluations	-0.207	-2.279	0.025	-0.164~-0.017	

Interpersonal relationships^b^	Asociality	-0.540	-6.817	0.000	-1.337~-0.734	0.423
	Negative self-evaluations	-0.262	-3.309	0.001	-0.340~-0.085	

Social participation^c^	Asociality	-0.495	-5.637	0.000	-1.709~-0.819	0.237

Recreation^d^	Avolition	-0.353	-3.417	0.000	-1.633~-0.433	0.402
	Asociality	-0.325	-3.152	0.000	-1.459~-0.332	

Self-reliance and execution^f^	SCoRS	-0.370	-3.645	0.000	-0.434~-0.128	0.402
	Avolition	-0.340	-3.354	0.001	-1.784~-0.458	

Employment^g^	Avolition	-0.604	-5.635	0.000	-1.51~-0.794	0.589
	SCoRS	-0.371	-4.004	0.002	-0.176~-0.040	
	Alogia	-0.269	-2.933	0.004	-0.464~-0.089	
	Anhedonia	-0.267	-2.847	0.005	-0.359~-0.064	
	Asociality	-0.253	-2.609	0.011	-0.581~-0.079	

^a^Final *F* = 20.36, *p* < 0.01. Variables removed (*p* = during multiple regression analysis). SCoRS *p* = 0.648; BPRS *p* = 0.344; negative other–self evaluations *p* = 0.086; anhedonia *p* = 0.515; avolition *p* = 0.241; blunted affect *p* = 0.450; alogia *p* = 0.826. ^b^Final *F* = 21.01, *p* < 0.01. Variables removed (*p* = during multiple regression analysis). SCoRS *p* = 0.608; BPRS *p* = 0.570; negative other–self evaluations *p* = 0.102; anhedonia *p* = 0.437; avolition *p* = 0.105; blunted affect *p* = 0.729; alogia *p* = 0.532. ^c^Final *F* = 22.46, *p* < 0.01. Variables removed (*p* = during multiple regression analysis). SCoRS *p* = 0.502; BPRS *p* = 0.349; negative self-evaluations *p* = 0.504; anhedonia *p* = 0.433; avolition *p* = 0.086; blunted affect *p* = 0.537; alogia *p* = 0.253. ^d^Final *F* = 41.83, *p* < 0.01. SCoRS *p* = 0.868; BPRS *p* = 0.169; negative self-evaluations *p* = 0.785. Variables removed (*p* = during multiple regression analysis). Asocialty *p* = 0.170; blunted affect *p* = 0.970; alogia *p* = 0.992. ^e^Final *F* = 69.39, *p* < 0.01. Variables removed (*p* = during multiple regression analysis). BPRS *p* = 0.502; negative self-evaluations *p* = 0.121; negative other–self evaluations *p* = 0.238; anhedonia *p* = 0.830; asocialty *p* = 0.333; avolition *p* = 0.698; blunted affect *p* = 0.760; alogia *p* = 0.669. ^f^Final *F* = 28.48, *p* < 0.01. Variables removed (*p* = during multiple regression analysis). BPRS *p* = 0.632; negative self-evaluations *p* = 0.149; anhedonia *p* = 0.170; asocialty *p* = 0.440; blunted affect *p* = 0.649; alogia *p* = 0.804. ^g^Final *F* = 28.75, *p* < 0.01. Variables removed (*p* = during multiple regression analysis). BPRS *p* = 0.642; negative self-evaluations *p* = 0.366; asocialty *p* = 0.501; blunted affect *p* = 0.625. SCoRS: Schizophrenia Cognition Rating Scale; CI: confidence interval; Adj: adjusted.

## Data Availability

The data used to support the findings of this study are restricted by the Institutional Review Board of the International University of Health and Welfare in order to protect patient privacy. Data are available from the corresponding author for researchers who meet the criteria for access to confidential data.
